# Identifying mitotane-induced mitochondria-associated membranes dysfunctions: metabolomic and lipidomic approaches

**DOI:** 10.18632/oncotarget.18968

**Published:** 2017-07-04

**Authors:** Ségolène Hescot, Larbi Amazit, Marie Lhomme, Simon Travers, Anais DuBow, Stephanie Battini, Geoffrey Boulate, Izzie Jacques Namer, Anne Lombes, Anatol Kontush, Alessio Imperiale, Eric Baudin, Marc Lombes

**Affiliations:** ^1^ INSERM UMR-S 1185, Le Kremlin-Bicêtre, France; ^2^ Endocrine Oncology, Gustave Roussy, Villejuif, France; ^3^ Institut Biomédical de Bicêtre, UMS-32, Le Kremlin Bicêtre, France; ^4^ ICANalytics, UMR-ICAN 116, University Pierre et Marie Curie, Paris, France; ^5^ ICube, UMR 7357, University of Strasbourg/CNRS and FMTS, Faculty of Medicine, Strasbourg, France; ^6^ Biophysics and Nuclear Medicine, University Hospital of Strasbourg, Strasbourg, France; ^7^ INSERM UMRS 1016, Institut Cochin, Paris, France; ^8^ Assistance Publique, Hôpitaux de Paris, Groupe Hospitalier Pitié-Salpétrière, Paris, France; ^9^ Assistance Publique, Hopitaux de Paris, Hopital Bicêtre, Department of Endocrinology, Le Kremlin-Bicêtre, France

**Keywords:** adrenocortical carcinoma, mitotane, mitochondria-associated membranes, molecular target, lipidomics

## Abstract

Mitotane (o,p’DDD), the most effective drug in adrenocortical carcinoma, concentrates into the mitochondria and impacts mitochondrial functions. To address the molecular mechanisms of mitotane action and to identify its potential target, metabolomic and lipidomic approaches as well as imaging analyses were employed in human adrenocortical H295R cells allowing identification of Mitochondria-Associated Membranes dysfunction as a critical impact of mitotane. Study of intracellular energetic metabolites by NMR spectroscopy showed that mitotane significantly decreased aspartate while concomitantly increased glutamate content in a time- and concentration-dependent manner. Such alterations were very likely linked to the previously described, mitotane-induced respiratory chain defect. Lipidomic studies of intracellular and intramitochondrial phospholipids revealed that mitotane exposure markedly reduced the phosphatidylserine/phosphatidylethanolamine ratio, indicative of a dysfunction of phosphatidylserine decarboxylase located in Mitochondria-Associated Membranes. Expression levels of Mitochondria-Associated Membranes proteins phosphatidylserine decarboxylase, DRP1, ATAD3A or TSPO were greatly reduced by mitotane as assessed by western blot analyses. Mitotane exposure markedly altered endogenous Mitochondria-Associated Membranes integrity and reduced the magnitude of mitochondria and the endoplasmic reticulum interactions as demonstrated by high resolution deconvolution microscopy and quantification. Finally, we showed that PK11195, a pharmacological inhibitor of the cholesterol translocator TSPO, embedded in Mitochondria-Associated Membranes, exerts a synergetic effect with mitotane in inducing Mitochondria-Associated Membranes disruption, apoptosis and in inhibiting steroid secretion. Altogether, our results demonstrate Mitochondria-Associated Membranes dysfunction in H295R cells treated with mitotane and that TSPO inhibition significantly potentiates mitotane antitumoral and antisecretory actions *in vitro*. This constitutes a potential and promising pharmacological strategy for patients with adrenocortical carcinoma.

## INTRODUCTION

Mitotane (o,p’DDD) is the only drug approved for the treatment of metastatic adrenocortical carcinoma (ACC) [1] but its molecular mechanism of action still remains to be elucidated. Maintaining plasma mitotane levels within the therapeutic window target of 8 to 30 mg/L represents the only predictive marker of antitumor response to date [2]. Therefore, identification of mitotane molecular targets would help therapeutic management of ACC patients.

Several studies have reported that mitotane impacts several key mitochondrial functions [3, 4]. We previously reported that therapeutic concentrations of o,p’DDD (50 μM, corresponding to approximately 14 mg/L in plasma) impaired the expression of several steroidogenic enzymes but also induced a selective and marked inhibition of mitochondrial respiratory chain complexes I and IV and a fragmentation of the mitochondrial network in human adrenocortical H295R cells [3]. Poli *et al*, subsequently showed that mitotane inhibited the expression of voltage-dependent anion channel (VDAC), a protein anchored in the outer mitochondrial membrane [4].

Recently, Sterol-O-Acyl/Acyl-coenzyme A Transferase 1 (*SOAT1*/ACAT1) was described as a new potential target of mitotane [5]. In this study, Sbiera *et al*, hypothesized that mitotane induced endoplasmic reticulum (ER) stress through ACAT1 inhibition leading to cell apoptosis.

Mitochondria-associated membranes (MAM) are subcellular structures belonging to the ER and are reversibly tethered to the mitochondria. MAM have been recently extensively studied [6] and constitute pivotal intracellular structures controlling key cellular processes such as apoptosis, calcium homeostasis, phospholipid metabolism, mitochondrial function, cholesterol metabolism and steroid synthesis, notably in adrenocortical cells [7]. Several marker proteins are enriched in MAM, among them VDAC, mitochondrial fusion-fission partners (mitofusin2, dynamin-1-related protein (DRP1)) and metabolic enzymes such as phosphatidylethanolamine-N-methyltransferase, phosphatidylserine synthases [8–10]. These later enzymes transfer phosphatidylserine (PS) from the ER to the mitochondria leading to phosphatidylethanolamine (PE) synthesis catalyzed by phosphatidylserine decarboxylase (PISD), an enzyme anchored in the inner mitochondrial membrane [11]. Tasseva *et al,* reported that an inhibition of PISD by siRNA strategy induced a selective defect in respiratory chain complexes I and IV accompanied with a mitochondrial fission [12]. These alterations were highly reminiscent of the mitotane-induced mitochondrial dysfunction, previously reported [3]. Moreover, other MAM's proteins such as ACAT1 or Steroidogenic Acute Regulatory protein (StAR) also play a major role in the metabolism and import of cholesterol into the mitochondria for steroidogenesis [6, 8, 9, 13] and consequently are highly relevant for steroid-producing adrenocortical cells. Of particular interest, it has been recently demonstrated that the acyl-CoA binding domain 3 (ATAD3A) protein, anchored in the inner mitochondrial membrane, acts as a scaffold protein, driving the formation of MAM and thus facilitating the transfer of cholesterol from the ER to the mitochondria [14]. In mitochondria, cholesterol reaches mitochondrial enzyme CYP11A1 with the help of the transduceosome, a multiprotein complex described by Papadopoulos *et al.,* that contains the translocator protein/peripheral-type benzodiazepine receptor or sigma 1 receptor (TSPO), StAR, ATAD3A and VDAC [15]. Taken together, these results stimulate the search for a specific target of mitotane related to or associated with MAM functions.

We hypothesize that MAM could be the targeted molecular complex of mitotane's action. In this present study, several preclinical *in vitro* studies including metabolomic and lipidomic approaches as well as apoptosis and steroidogenic production assessments and cell imaging indicate that MAM formation and function are markedly inhibited by mitotane exposure, synergistically with the concomitant use of a TSPO inhibitor.

## RESULTS

### Metabolomics

**Figure 1:**
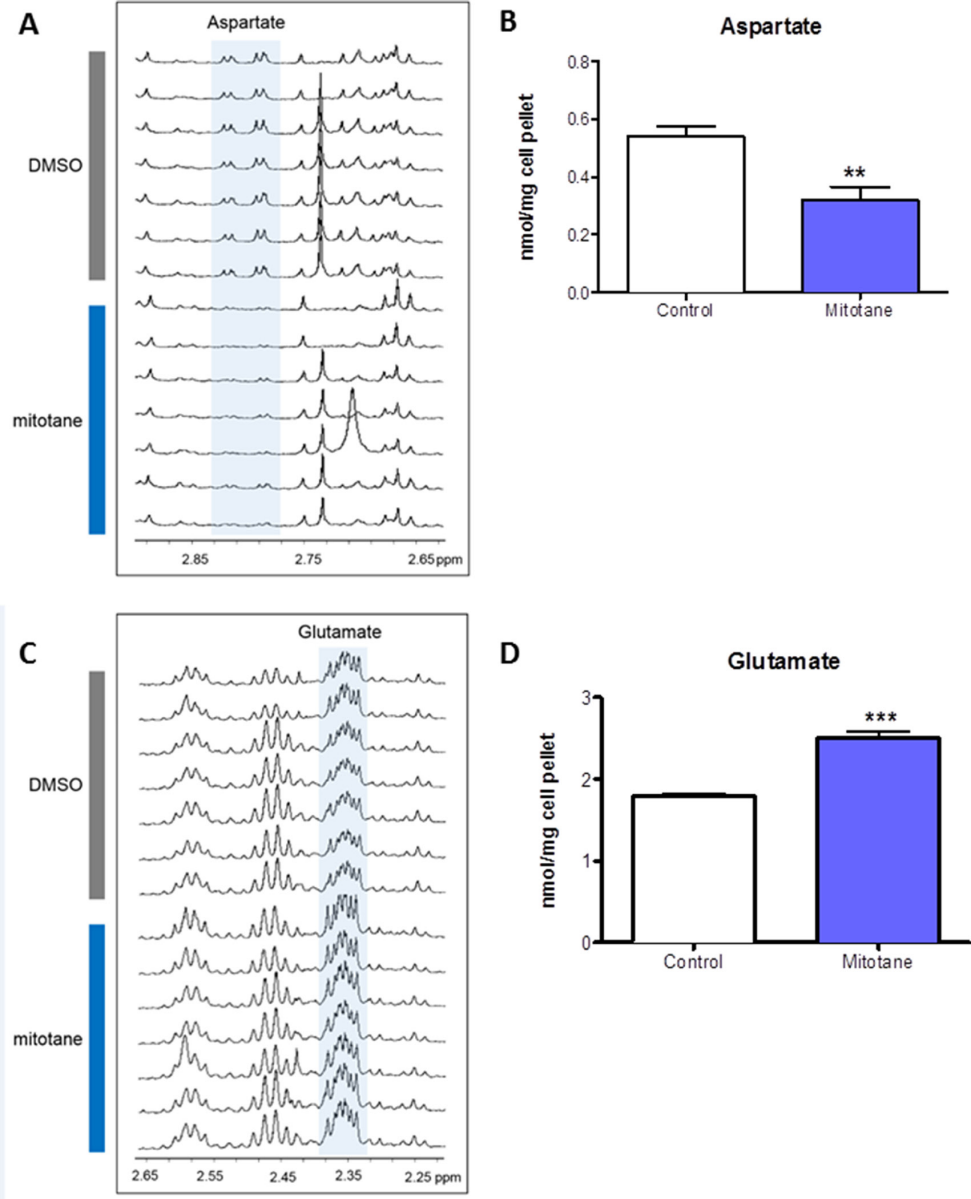
Metabolome of H295R cells H295R cells were treated with 50 μM mitotane for 48 h. (**A**) and (**C**) 1D ^1^H-HRMAS spectra of untreated and mitotane-treated human adrenocortical H295R cells. (**B**) Aspartate measurements in whole cell extracts of untreated or mitotane-treated H295R cells. (**D**) Glutamate measurements in whole cell extracts of untreated or mitotane-treated H295R cells. Results are means ± SEM of 6 different experiments and are expressed in nmol/mg of cell pellet. **P < 0.01 and ***P < 0.001, Mann-Whitney *U*-tests. Mitotane decreases aspartate and increases glutamate levels in H295R cells.

**Figure 2:**
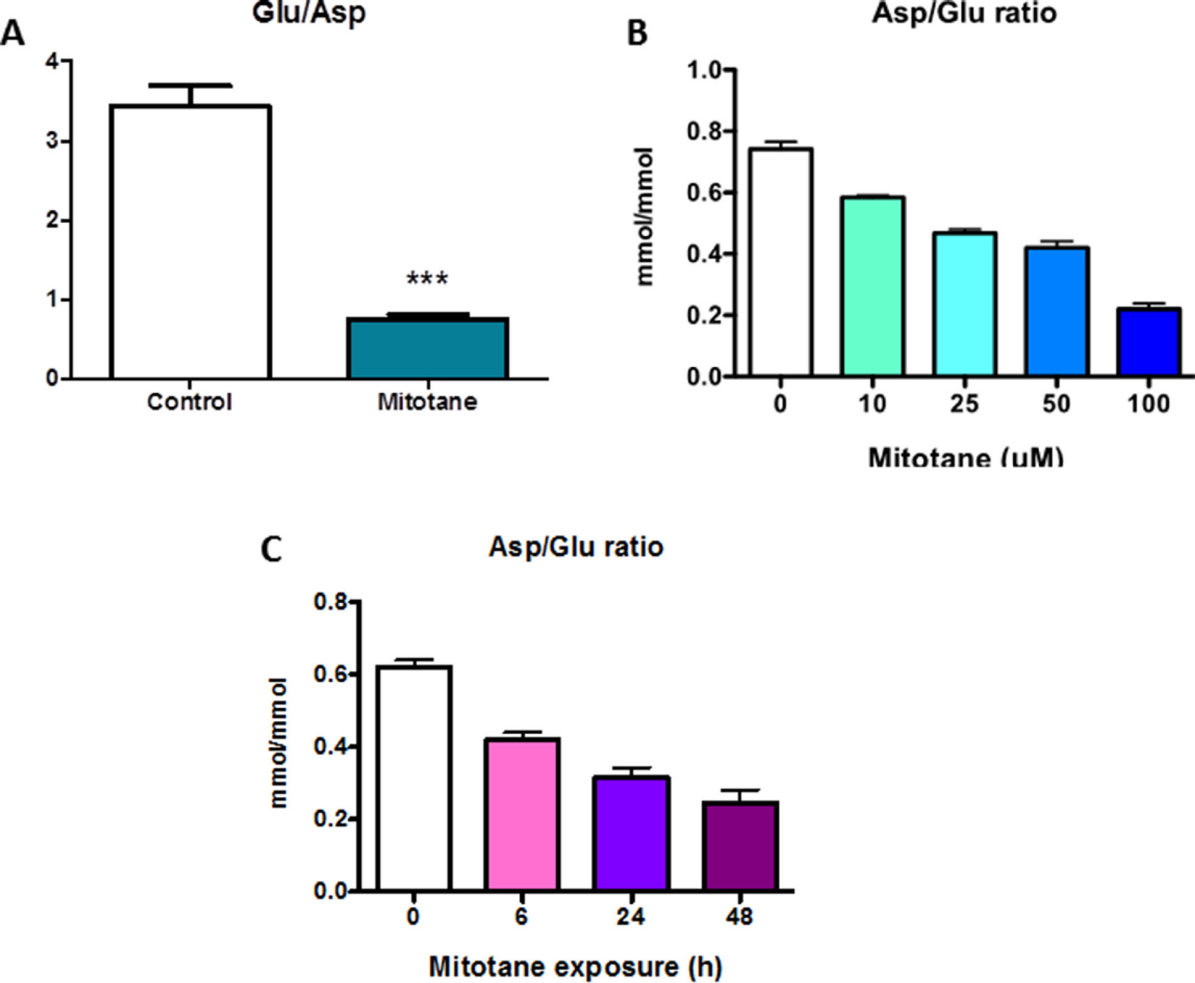
Metabolomic specific hallmark of mitotane H295R cells were treated with 50 μM for 48 h (**A**) Results are means ± SEM of 6 different experiments. ***P < 0.001, Mann-Whitney *U*-tests.with increasing concentrations of mitotane (0 to 100 μM) for 48 h (**B**) or with 50 μM mitotane for various periods of time (0 to 48 h) (**C**). Results are means ± SEM of 3 to 5 different experiments. *P < 0.05 and **P < 0.01, Kruskal-Wallis tests. Mitotane induces a decrease of Asp/Glu ratio. Asp: aspartate, Glu: glutamate.

We used HRMAS NMR spectroscopy to evaluate the impact of mitotane on the metabolome of tumoral cells. At visual inspection, all the spectra obtained from the 14 analyzed cellular samples were of good quality. The generated multivariate two-component PLS-DA allowed a clear separation of untreated from mitotane-treated H295R cells showing accurate representation of the data and a good cumulative confidence criterion of fit (R2Y = 0.87) and prediction (Q2 = 0.56). According to spectra visual evaluation and the results of PLS-DA model, both aspartate and glutamate were the most discriminant metabolites between cellular subgroups. As illustrated in Figure 1A, HRMAS spectra of untreated or mitotane-treated H295R cells demonstrated that aspartate content was drastically reduced upon mitotane exposure. Further quantification indicated a significant reduction in the amount of aspartate after mitotane treatment from 0.54 ± 0.04 to 0.32 ± 0.04 nmoL/mg cell pellet (*P* < 0.01, *n* = 6) (Figure 1B). Concomitantly, glutamate content significantly increased in mitotane-treated cells from 1.80 ± 0.03 to 2.51 ± 0.08 nmoL/mg cell pellet (*P* < 0.001, *n* = 6) (Figure 1C–1D). Mitotane therefore induced a robust decrease in intracellular aspartate/glutamate ratio (Figure 2A). The time- and dose-dependent decrease of aspartate/glutamate ratio showed that this metabolomic hallmark could be considered as a specific index of mitotane action (Figure 2B). This finding was consistent with the previously identified mitochondrial respiratory chain defect, selectively targeting complex I and complex IV enzymatic activities [3]. Moreover, aspartate biosynthesis was recently shown to play a crucial role in cell proliferation in the presence of respiratory chain defect [16]. Thus, the defect in oxidation of NADH by complex I causes major dysfunction of the tricarboxylic acid cycle because of the depletion of the intracellular content of NAD^+^. Such a metabolomic hallmark strongly suggests that mitotane should exert major effects in terms of metabolic adaptations and lipid homeostasis.

### Impact of mitotane on phospho- and sphingolipids

**Figure 3:**
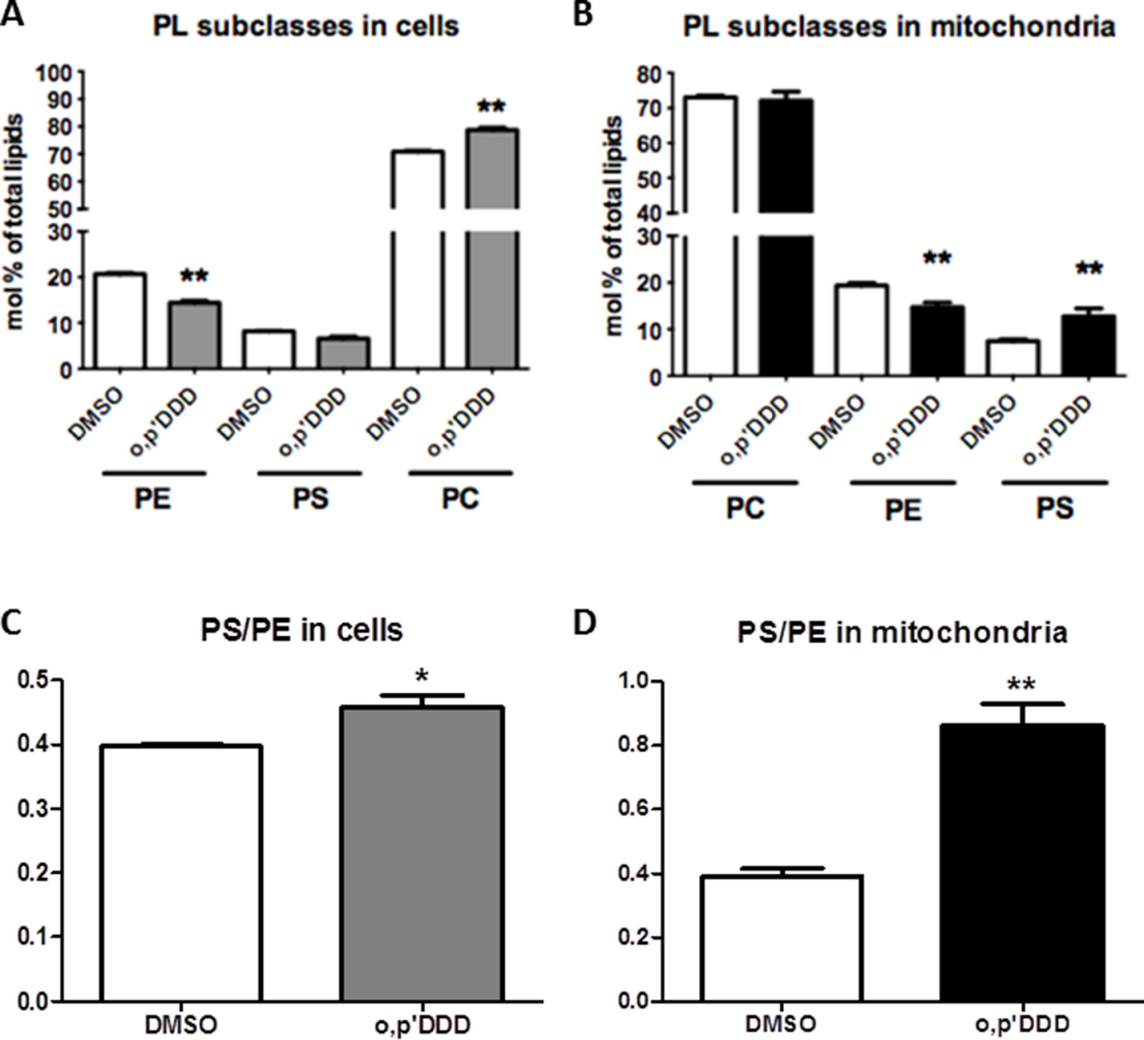
Lipidomic analyses of H295R cells and the corresponding mitochondrial fractions isolated as described in Materials and Methods section H295R cells were treated with 50 μM for 48 h. PC: phosphatidylcholine, PE: phosphatidylethanolamine, PS: phosphatidylserine. (**A**) and (**B**) Levels of PC, PE and PS in H295R whole cells and isolated mitochondria expressed as molar percentage of total lipids. (*n* = 6). (**C**) and (**D**) Molar ratio of PS/PE in H295R cells and isolated mitochondria. Results are means ± SEM of 6 different experiments. *P < 0.05, **P < 0.01 and ***P < 0.001, Mann-Whitney *U*-tests. Mitotane increases PS/PE ratio.

**Figure 4:**
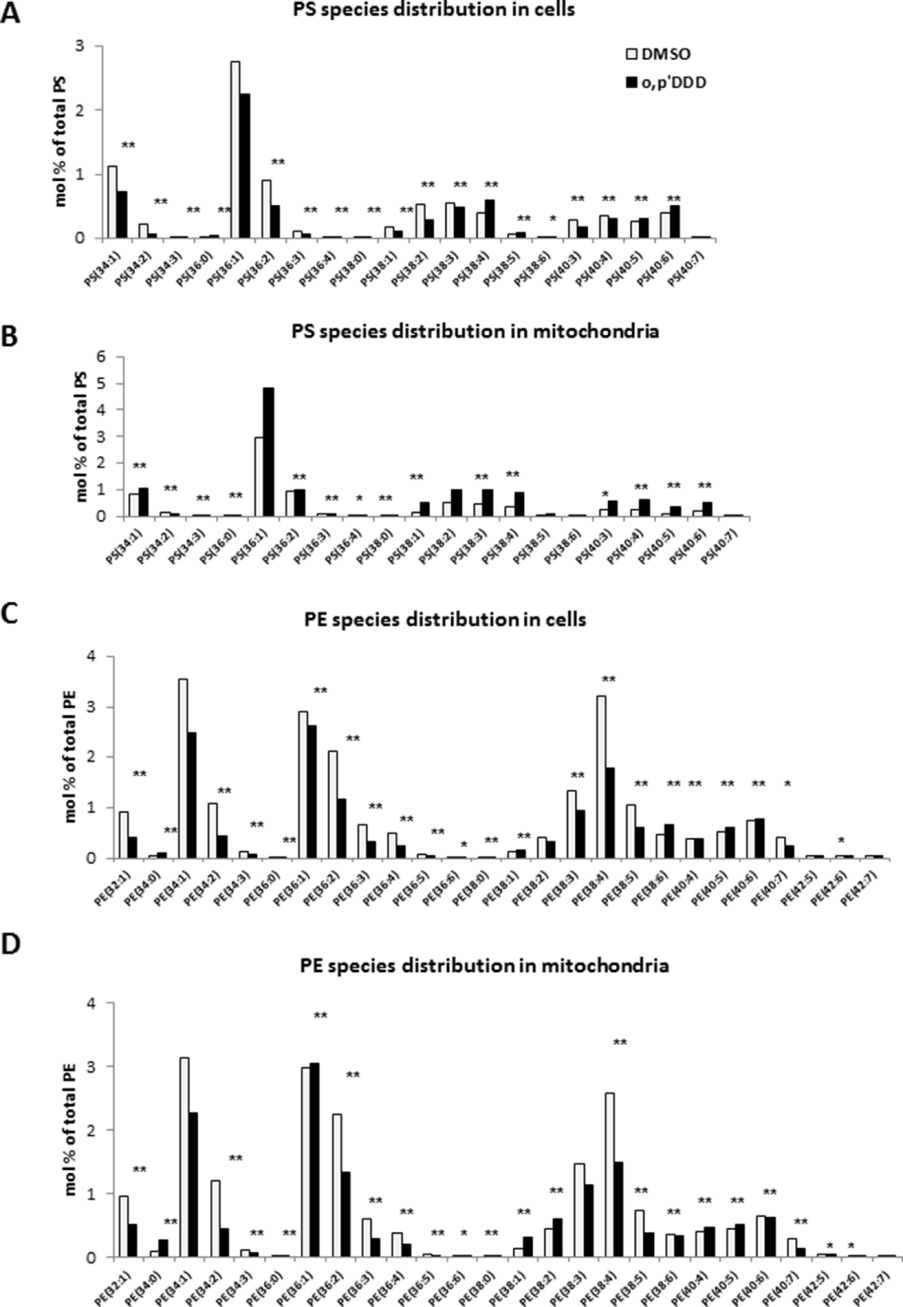
(**A**) and (**C**) PE and PS species distribution in cells expressed as molar percentage of total PE and PS. (**B**) and (**D**) PE and PS species distribution in mitochondria expressed as molar percentage of total PE and PS. *P < 0.05 and **P < 0.01, Mann-Whitney *U*-tests. Results are means ± SEM of 6 different experiments. H295R cells were treated with 50 μM for 48 h. PE: phosphatidylethanolamine; PS: phosphatidylserine.

To further explore the impact of mitotane on lipid metabolism, we performed lipidomic analyses on both whole cells and isolated mitochondrial fractions. Phospholipids and sphingolipids species were studied in H295R cells before and 48 h after treatment with 50 μM mitotane in several independent experiments. Although differences in lipid profiles limited pooling the data, results showed a decrease in PE content in whole H295R cells (Figure 3A). The phospholipid alterations associating increased PS content and decreased PE content were much more pronounced in isolated mitochondria where differences reached statistical significance (Figure 3B). The PS/PE ratio significantly increased in whole cells and isolated mitochondria after mitotane treatment (Figure 3C–3D), a result consistent with a defective activity of phosphatidylserine decarboxylase (PISD), an enzyme localized in the inner mitochondrial membrane and involved in PE synthesis. In addition, changes in the relative distribution of phospholipid species according to the length and the degree of saturation of fatty acid moieties were observed (Figure 4). For instance, mitotane induced significant increase in mitochondrial PS/PE 36:2, 38:4 and 40:6 ratios. Of particular interest, analyses of the whole lipid species did not reveal any significant effect on sphingolipids (including sphingomyeline and ceramides) but showed an accumulation of PS precursors (phosphatidic acid and phosphatidylcholine), suggesting that mitotane induced a selective alteration of the phospholipid metabolism.

### Impact of mitotane on MAM proteins

Lipidomic changes were consistent with a mitotane-induced defective activity of PISD, an enzyme of PE synthesis localized in the inner mitochondrial membrane and likely involved in MAM. Furthermore, the fragmentation of the mitochondrial network, previously observed in mitotane- treated H295R cells [3], suggested that proteins involved in the mitochondrial fission/fusion equilibrium, notably DRP1, another player of MAM, might be inhibited by mitotane treatment. Based on these observations, we hypothesized that mitotane could modify MAM formation and function and evaluated the impact of mitotane on steady state levels of several MAM protein components.

**Figure 5:**
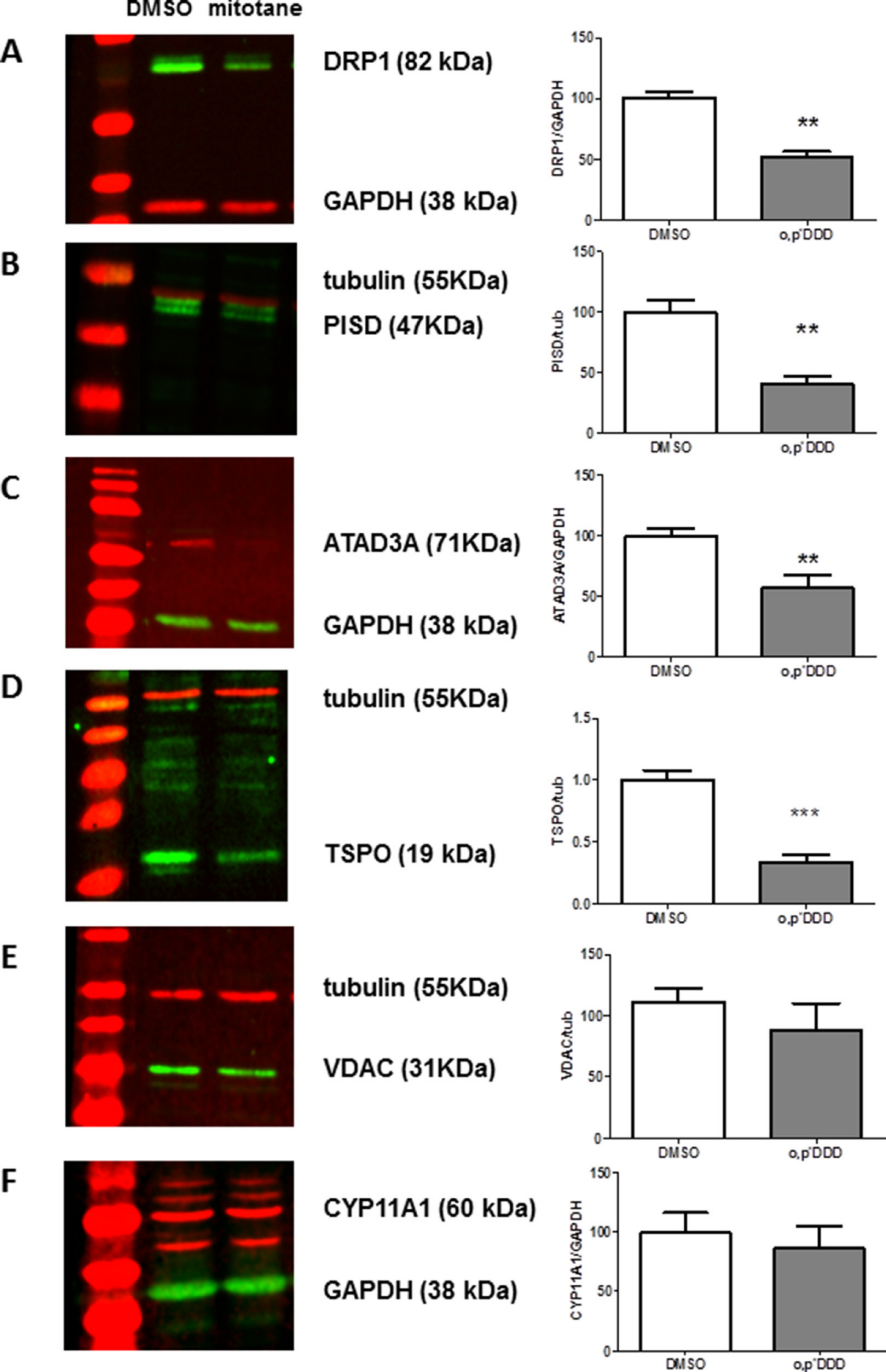
Expression of MAM components by Western Blot analyses H295R cells were treated with 50 μM mitotane for 48 h. Steady-state levels and relative expression of DRP1 (**A**), PISD (**B**), ATAD3A (**C**), TSPO (**D**), VDAC (**E**) and CYP11A1 (**F**) proteins by Western Blot. Representative images (left panels) and quantification of band intensities (right panels) corresponding to the ratio between the gene product of interest and the corresponding loading controls (Tubulin or GAPDH) are shown. Results are means ± SEM of 3 to 4 different experiments. **P < 0.01, Mann-Whitney *U*-tests. Mitotane inhibits protein expression of some resident proteins of MAM: DRP1, PISD, TSPO and ATAD3A, but not all (no effect on VDAC and CYP11A1 expression).

Both PISD and DRP1 protein steady state levels were markedly decreased in H295R cells treated by 50 μM mitotane for 48 h (Figure 5A–5B). This was also the case for ATAD3A, the scaffold-protein that links ER to mitochondria (Figure 5C) as well as for TSPO protein (Figure 5D). In contrast, VDAC1 (the voltage-dependent anion channel), and CYP11A1 protein expression were not significantly reduced by mitotane (Figure 5E–5F) under our experimental conditions.

### Morphological integrity of MAM upon mitotane exposure

**Figure 6:**
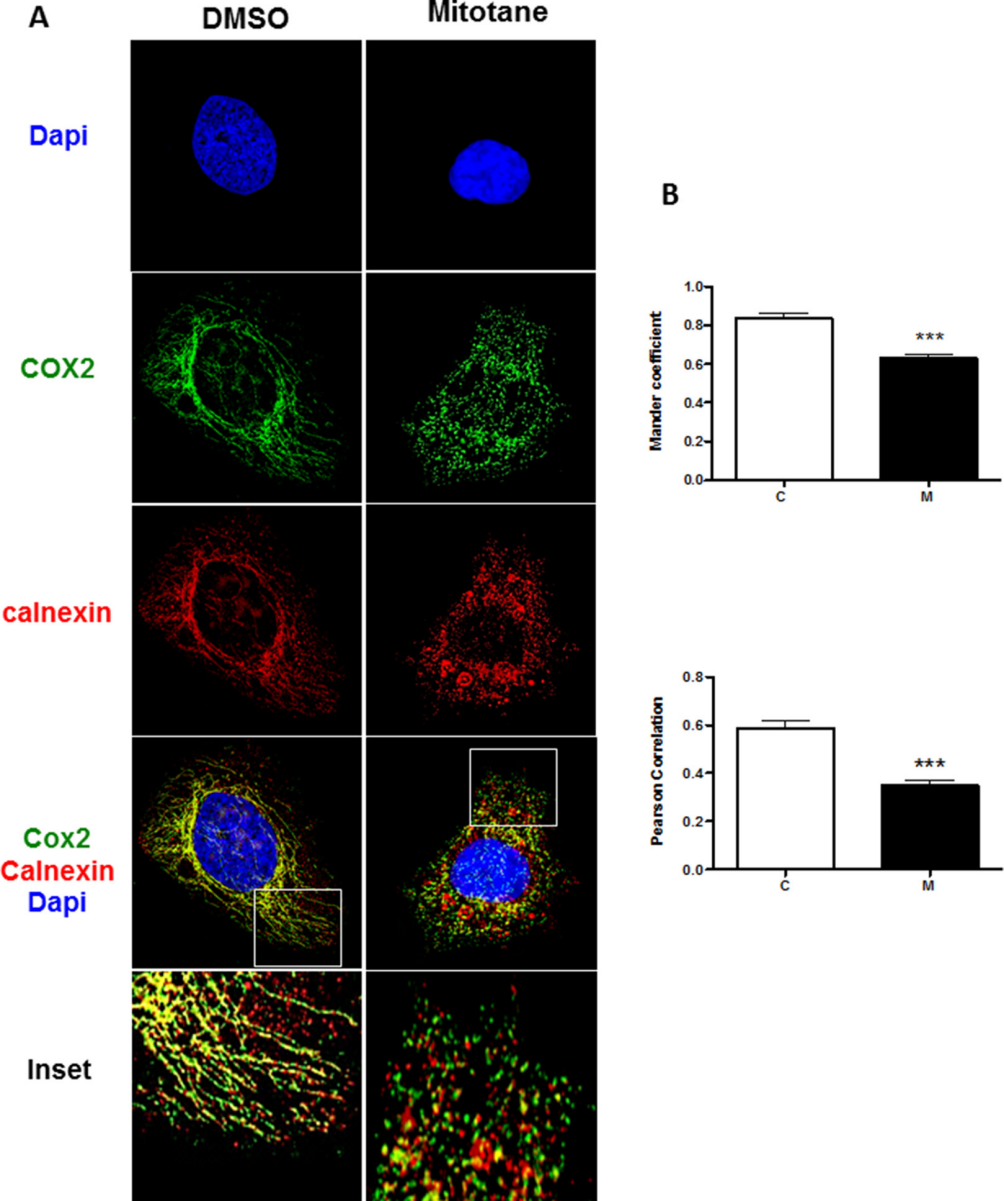
Mitotane reduces the integrity and extent of mitochondria-associated membranes in human adrenocortical carcinoma cells (**A**) H295R cells, incubated for 48 h with 0.05% v/v DMSO (control) or 50 μM mitotane, were fixed and analyzed by immunocytochemistry as described in Materials and Methods section. The anti-Calnexin antibody (Red staining) and the anti-COX2 antibodies (Green staining) were used to detect the endogenous endoplasmic reticulum and mitochondria, respectively (see Materials and Methods section). DAPI staining delineates the nuclei. A z-series of focal planes was digitally imaged and deconvolved with a 3D blind iterative algorithm to generate high-resolution images. The inset shows a magnification of the selected cytosolic region (White Square). Note the prominent colocalization of COX2 and calnexin in the control experiment (DMSO, upper panels) and the disruption of this colocalization in the presence of 50 μM mitotane (right panels). (**B**) Manders' Colocalization Coefficient (MCC) and Pearson Correlation Coefficient (PCC) quantification, are shown corresponding to the colocalization of the COX2 and Calnexin for each condition, DMSO (C) and mitotane (M), respectively (*n* > 20). ***P < 0.001, Mann-Whitney *U*-tests.

To investigate whether mitotane affects the number of ER-mitochondria contacts in H295R cells, we analyzed the colocalization of the endogenous Ca^2+^-binding and quality control chaperone calnexin known to reside at MAMs [17, 18] and the mitochondrial subunit 2 of the cytochrome c oxidase, COX2, which is exposed to the mitochondrial intermembrane space. Under basal conditions i.e. in the absence of treatment, and as anticipated [3], the mitochondrial compartment appeared as a highly filamentous, interconnected tubular network (Figure 6A). Calnexin signal (red staining) clearly overlapped with COX2 signal (green staining) along the mitochondrial network as shown in merged images (yellow staining) and also presented in zoomed inset microphotography (right lower panels). In sharp contrast, after 50 μM mitotane exposure, the mitochondrial compartment exhibited dramatic fragmentation towards punctiform pattern (Figure 6A). This fragmentation was accompanied by a reduction of the colocalization of calnexin with COX2, both calnexin and COX2 signals appearing most often juxtaposed rather strictly colocalized, as illustrated in Figure 6A (right lower panel). In order to validate these observations, we also performed the quantification of this colocalization in multiple cells and both the Manders' Colocalization Coefficients (MCC) and the Pearson Correlation Coefficient (PCC) were calculated for each condition [19]. Cells that were selected did not undergo apoptosis and showed normal cellular/nuclear morphological integrity (i.e., without DNA condensation, cell blebbing, nuclear shrinkage or cell rounding). Our results demonstrate that mitotane significantly reduces the fraction of COX2 that colocalizes with Calnexin (Figure 6B, Manders' and Pearsons’ coefficients). Taken together, these data indicate that mitotane exposure markedly alters the endogenous MAM integrity and reduces the magnitude of organelle interactions between mitochondria and ER in human adrenocortical cells.

### Synergistic effects of mitotane and PK11195, a pharmacological inhibitor of TSPO

Despite recent contradictory data in mice, the 18 kDa translocator protein TSPO, previously known as a peripheral benzodiazepine receptor, was initially described to be involved in cholesterol transfer from the outer to the inner mitochondrial membrane [20]. Given the close structural similarity between the chemical structure of cholesterol, benzodipazepine compounds and mitotane, all harboring several aromatic cycles, we hypothesized that TSPO could be a potential target of mitotane. To further explore this possibility, we incubated H295R cells with a combination of both mitotane and PK11195, a high affinity and selective pharmacological inhibitor of TSPO, in order to check whether this antagonist may rather increase the efficiency of mitotane and induce mitochondrial fragmentation. Cells were analyzed by immunocytochemistry and colocalization between COX2 and Calnexin, reminiscent of MAM, was quantified in multiple cells by using PCC and MCC calculation.

**Figure 7:**
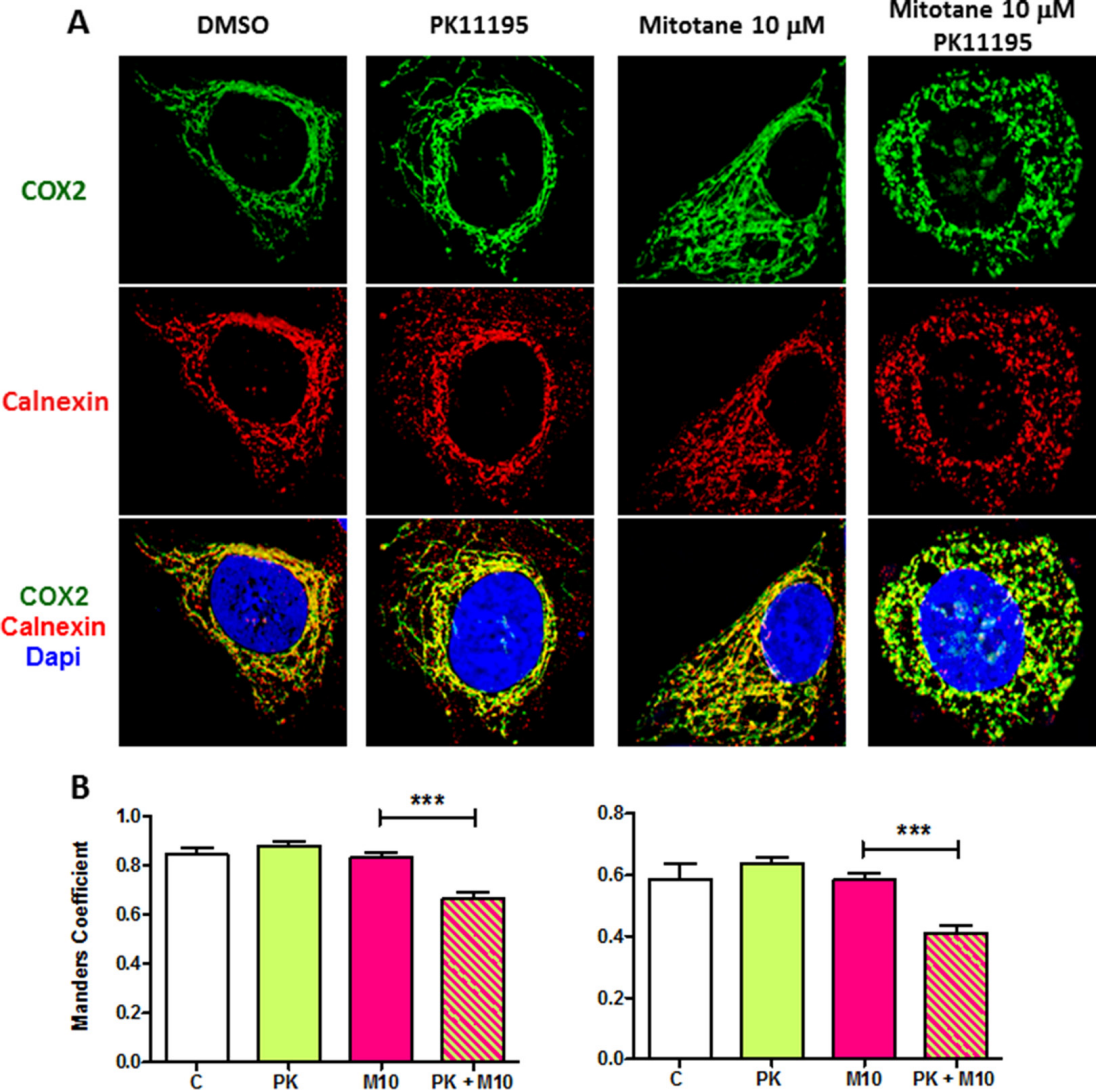
PK11195 potentiates mitotane-induced MAM disruption (**A**) H295R cells were incubated for 48 h with DMSO (0.1% v/v) or increasing concentrations of mitotane (from 10 to 50 μM) in the absence or presence of PK11195 (50 μM). After drug treatment, cells were fixed and analyzed by immunocytochemistry. The anti-COX2 (green) and anti-calnexin antibodies (red) were used to detect the mitochondria and ER, respectively. DAPI staining delineates the nuclei. A z-series of focal planes was digitally imaged and deconvolved with a 3D blind iterative algorithm to generate high-resolution images. In the absence of PK11195, the mitochondrial interconnected tubular network is readily disorganized in the presence of high concentrations of mitotane (50 μM) but not at lower concentrations (10 μM). The ER staining follows similar interconnected pattern. In sharp contrast, in the presence of PK11195, the mitochondrial filamentous aspect dramatically disappears and replaces by a punctiform network already visible in presence of the low 10 μM mitotane concentration. Merged images comfort MAM disruption and the potentiation of mitotane action by PK11195. (**B**) Manders' Colocalization Coefficient (MCC) and Pearson Correlation Coefficient (PCC) and quantification, are shown to quantify colocalization of the two proteins for each condition, DMSO (C) PK11195 (PK), mitotane 10 μM (M 10) and PK and mitotane (PK +M10), respectively. (*n* > 7). ***P < 0.001, Mann-Whitney *U*-tests.

**Figure 8:**
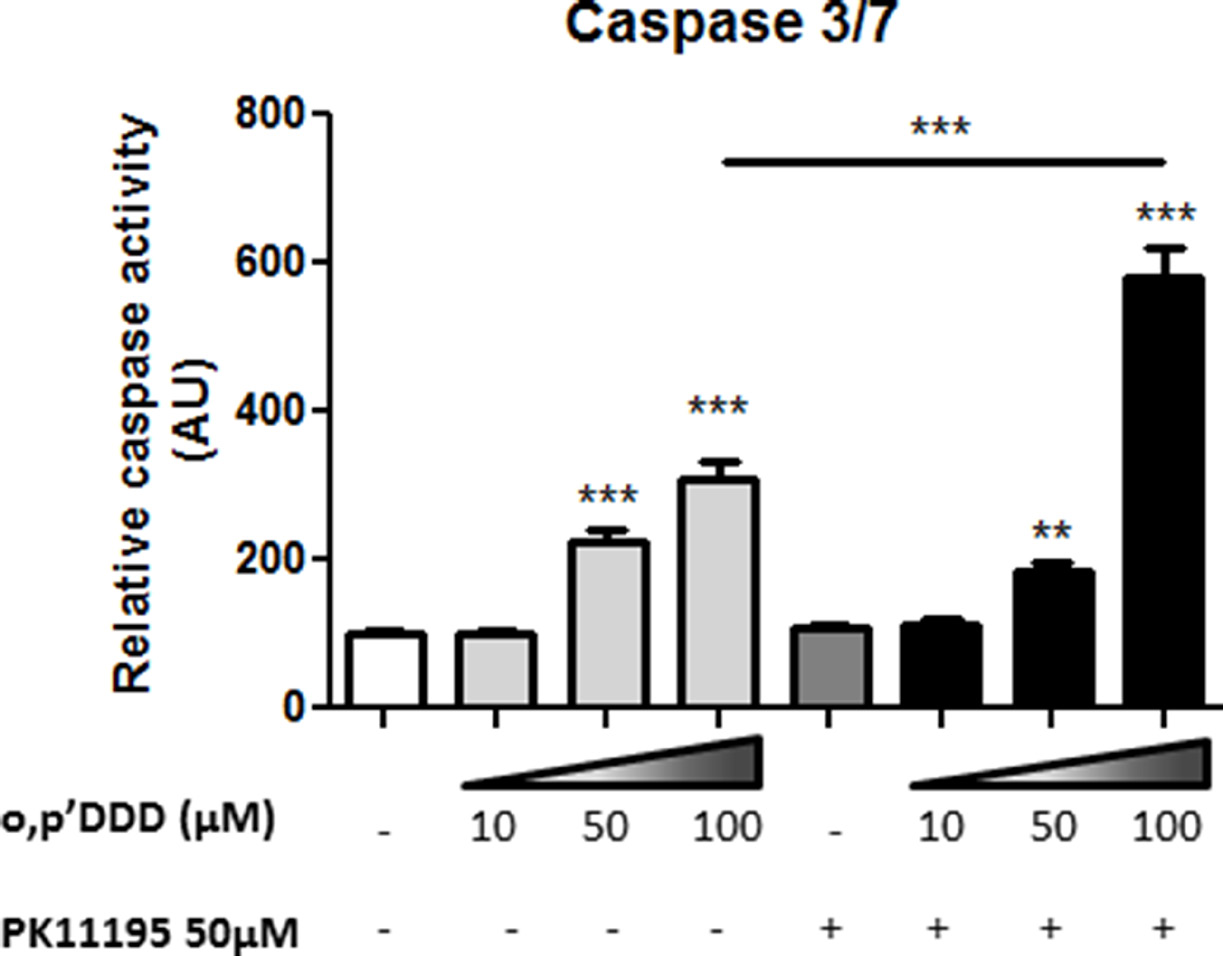
Synergistic effects of PK1195 and mitotane in inducting cell apopotosis H295R cells were treated with increasing concentrations of mitotane (0–100 μM) for 24 h in the absence or presence of 50 μM PK11195. Caspase activity was measured as described in Material and Methods section. Results are means ± SEM of 6 different experiments. ***P < 0.001, Mann-Whitney *U*-tests. Mitotane and PK11195 exert a synergetic effect in inducing apoptosis in H295R cells. Results are means ± SEM of 2 different experiments each performed in 8 determinations.

PK11195, at relatively low concentrations (50 μM) did not induce major morphological changes in the mitochondrial network of H295R cells nor decrease the colocalization with calnexin (Figure 7A and 7B). Likewise, a low concentration of 10 μM mitotane alone did affect neither mitochondrial network nor calnexin colocalization as shown in Figure 7A and 7B (Mitotane condition. In sharp contrast, a combination of the same concentration of mitotane in addition with 50 μM PK11195 dramatically induced a marked punctiform pattern of mitochondria as well as of the reticular ER network (Figure 7A Mitotane/PK11195 condition and Figure 7B. This was associated with statistically significant lower MCC and PCC value than that measured in control PK or Mitotane exposure alone (Figure 7B). In the presence of 50 μM mitotane, PK11195 could not further disrupt the mitochondrial network and MAM interactions (Supplementary Figure 2A and 2B). This result suggests that 50 μM mitotane is capable to induce a maximal disruption of mitochondria [3] and consequently that an efficient PK11195 synergistic effect is only visible at low concentration of mitotane. This phenomena was unconnected from an apoptosis process since we could neither observe nor measure any characteristic change in the nuclear and cellular morphology of co-treated cells (data not shown). Furthermore, given that MAM are major contributor regulating cellular homeostasis such as apoptosis, we also showed that the two compounds PK11195 and mitotane disclosed synergistic effects on apoptosis (Figure 8). PK11195 alone did not induce Caspase 3/7 activity as a marker of apoptosis in H295R cells whereas mitotane dose-dependently increases Caspase 3/7 activity. Combining 50 μM PK11195 and 10 μM of mitotane did not increase Caspase 3/7 activity thus excluding that MAM dysfunction upon mitotane exposure at a low concentration of 10 μM is a consequence of cell apoptosis. Combining 50 μM PK11195 and 100 μM mitotane significantly potentiates the activation of caspase 3/7 activity, consistent with a synergistic effect of both drugs on H295 cells.

**Figure 9:**
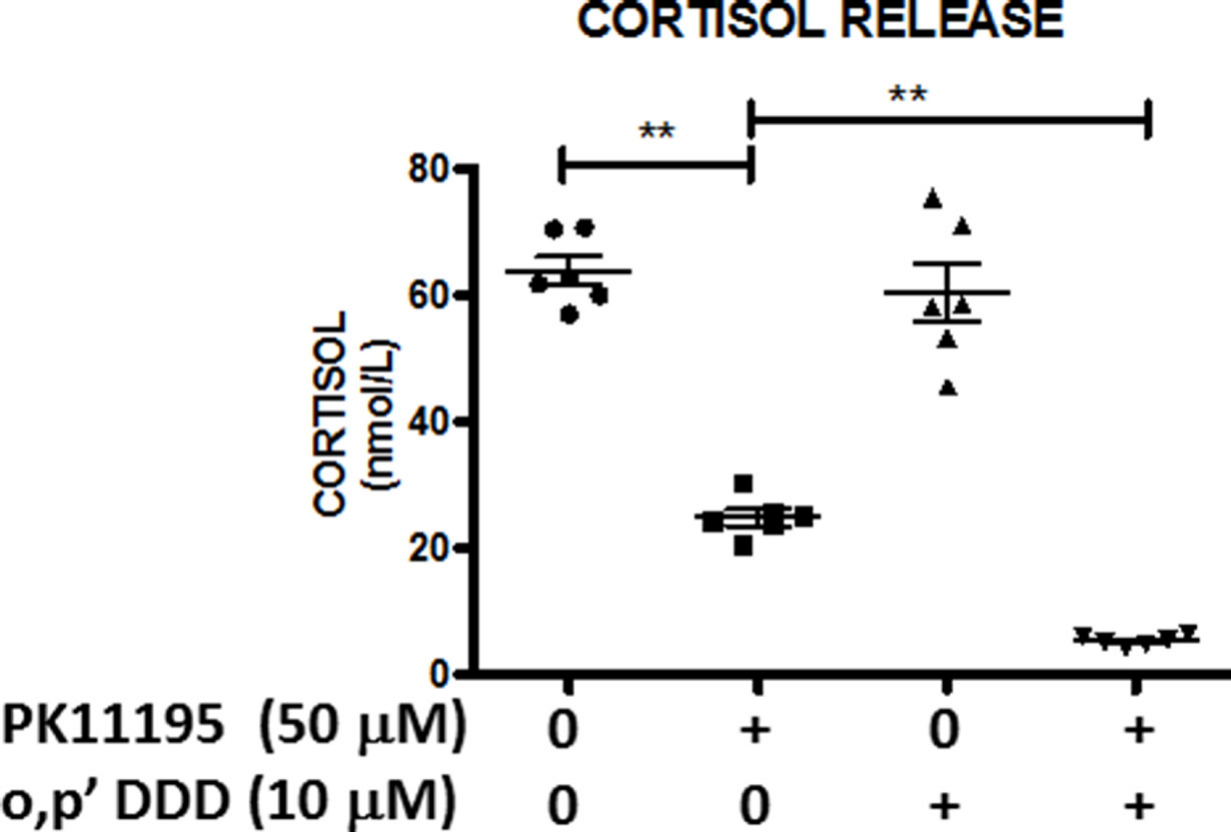
Antisecretory and synergistic effects of PK1195 and mitotane on cortisol release H295R cells were treated with low concentrations of mitotane (10 μM) for 48 h in the absence or presence of 50 μM PK11195. Cortisol concentrations (nmol/L) in the supernatants of these H295R cells were measured by LC-MS/MS and compared according to experimental conditions: DMSO (●); PK11195 50 μM (□); o,p’DDD 10 μM (▲) and PK11195 50 μM + o,p’DDD 10 μM (◆). Results are expressed as mean ± SEM and individual values presented. Means were compared using non-parametric Mann Whitney *U*-tests. **P < 0.01. Mitotane and PK11195 exert synergetic effects in inhibiting cortisol secretion in H295R cells.

It is well established that mitotane also exerts anti-secretory properties [3]. Using measurements of cortisol production in H295R cell supernatants with the highly specific and sensitive LC-MS/MS method, we finally demonstrated the synergy between PK11195 and mitotane on steroid secretion (Figure 9). While mitotane alone, at a low 10 μM concentration, did not significantly modify cortisol secretion, addition of PK11195 significantly reduced cortisol release by more than 80% as compared to PK11195 alone which also inhibited cortisol release. Collectively, these findings provided first evidence that o,p’DDD and TSPO inhibitor synergistically inhibited cortisol synthesis in human adrenocortical H295 cells.

## DISCUSSION

Mitotane remains the most effective treatment for ACC but its molecular mechanism of action is still unclear and its molecular target unknown. It has been previously demonstrated that mitotane has a marked mitochondrial impact [3, 4] but the drug also induces ER damages resulting in ER stress as recently shown [5]. In the present study, we identify MAM as one main intracellular target of mitotane action.

**Figure 10:**
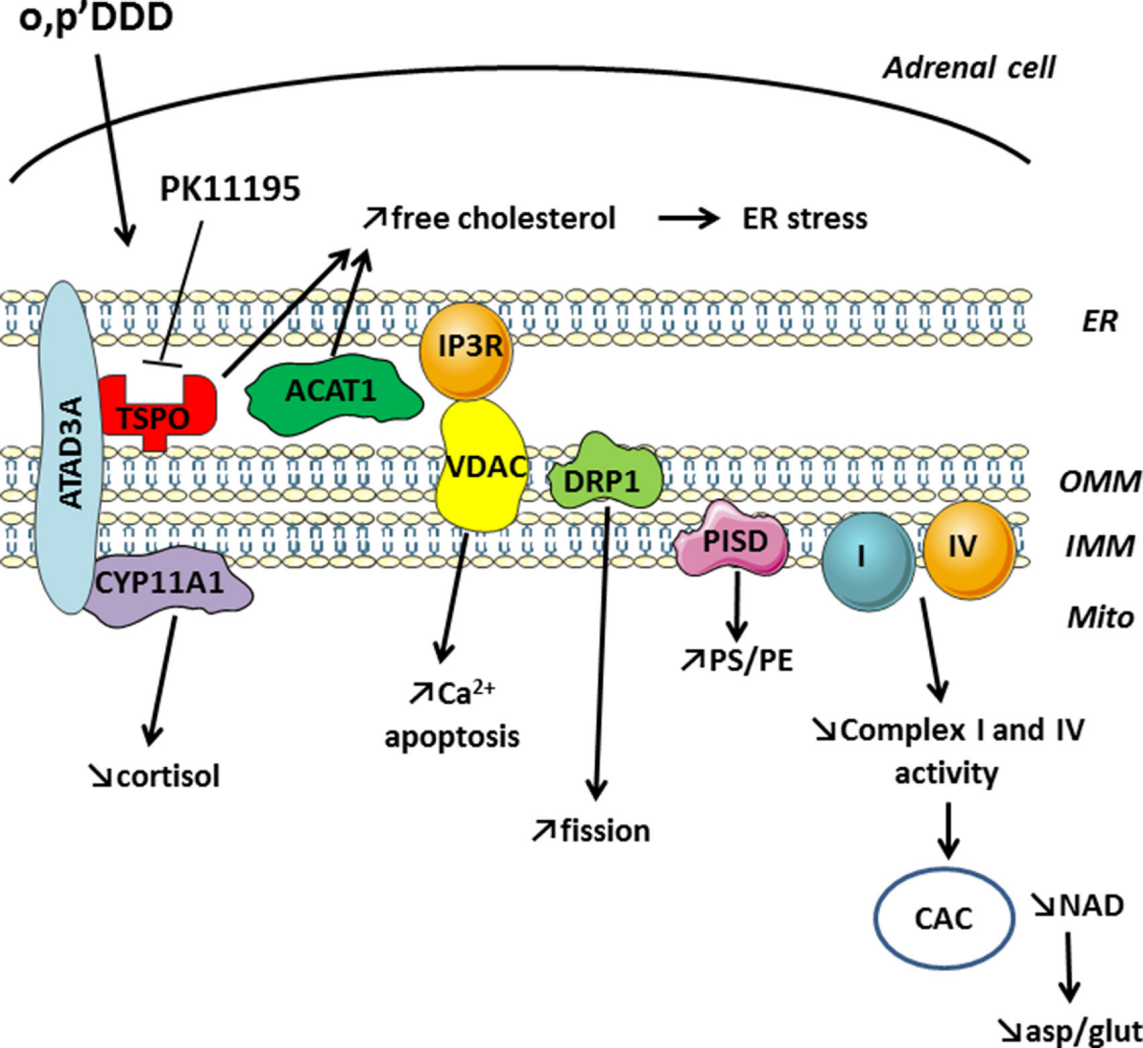
Mitotane action on structural and functional properties of MAM and metabolic consequences leading to cell death ER: endoplasmic reticulum; mito: mitochondria; PS: phosphatidylserine; PE: phosphatidylethanolamine; CAC: citric acid cycle; asp: aspartate; glu: glutamate.

We previously showed by immunocytochemistry that mitotane induces a fragmentation of the mitochondrial network and demonstrated that this was accompanied by a specific inhibition of respiratory chain complexes I and IV activities [3]. Recently, Tasseva *et al.*, observed that an inhibition of PISD expression in PSB-2 cells by siRNA was responsible for mitochondrial damages induced by low PE including inhibition of respiration, decrease in calcium uptake, and increase of mitochondrial fragmentation [12]. Because of these similar phenotypes, we studied the impact of mitotane on PISD with complementary approaches and confirmed that mitotane inhibits both PISD protein expression by Western Blot and function by lipidomic studies in human adrenocortical H295R cells. PISD is localized in MAM and the effects such as a mitochondrial fragmentation could be secondary to MAM disruption. Indeed, the mitochondrial network is dynamically regulated by fusion and fission events that involve different proteins including DRP1 (dynamin-related protein type 1), also component of MAM structure. Likewise, mitotane reduces DRP1 protein steady state levels in H295R cells. Surprisingly, PISD and DRP1 mRNA levels were significantly increased under identical mitotane incubation conditions (data not shown). Such a dissociation between mitochondrial protein expression and their corresponding transcription levels has already been reported for the mitochondrial toxicity of antiretroviral compounds in adipocytes [21]. Yet, the compensatory effect reported here for adrenocortical cells with mitotane remains to be further characterized. Our findings also demonstrated that mitotane directly inhibits the expression of some but not all structural MAM resident proteins. For instance, CYP11A1 or VDAC expression does not seem to be modified in a significant manner after 50 μM mitotane exposure for 48 h. However, a reduced VDAC protein expression has been previously demonstrated [4] and CYP11A1 expression decreased after longer mitotane exposure up to 72 h (data not shown). We showed using immunocytochemistry and high resolution deconvolution microscopy followed by quantification of Manders and Pearson colocalization coefficients that mitotane induces a disruption of MAM and its functional consequences are summarized in Figure 10. ER stress is induced by an increase in free cholesterol intracellular content as previously reported and could result from an inhibition of both TSPO and ACAT1, also localized in MAM [5]. Finally, the combination of the increase in intramitochondrial Ca^2+^ concentration together with a concomitant respiratory chain defect, leading to accelerated Reactive Oxygen Species (ROS) production, are responsible for cell apoptosis.

During the last decade, MAM were reported to be affected in Alzheimer disease [22]. The observed features including disruption of mitochondrial network and dysfunction in calcium, phospholipid and cholesterol metabolism are very similar to the effects demonstrated in H295R cells upon mitotane exposure. Interestingly, neurological side effects in patients treated with mitotane [23] resembled certain Alzheimer disease symptoms, providing support for a similar pathogenic mechanism.

We previously showed that intracellular mitotane massively enters into and concentrates in the mitochondrial compartment [24]. Two non-mutually exclusive hypotheses might account for a specific interaction of mitotane with MAM. First, mitotane, a highly lipophilic molecule, could enter directly into the MAM lipid membrane. Indeed, a recent work showed that mitotane inserts into the bilayer lipid membrane inducing disturbance and alterations in membrane integrity [25]. In this latter study, mitotane-induced alterations required the presence of PE which is a major component of the mitochondria membrane, synthesized by PISD [26]. Moreover, MAM lipid raft-like microdomains have been recently identified [27]. The second hypothesis relies on the fact that mitotane might bind TSPO, the transmembrane intramitochondrial cholesterol translocator protein, and may act as a mitochondrial benzodiazepine receptor antagonist, although not directly assayed in the present work. Indeed, mitotane is a highly lipophilic compound and displays two aromatic cycles, as do most TSPO ligands including benzodiazepine derivatives [28]. To further explore this hypothesis, H295R cells were co-incubated with mitotane and PK11195, a high affinity and selective pharmacological inhibitor of TSPO. We showed a synergistic effect between the two molecules in inducing MAM disruption and apoptosis as well as in inhibiting steroidogenesis in human adrenocortical cells. MAM disruption, but no apoptosis observed when cells were treated with 10 μM of mitotane. Thus, targeting TSPO on top of mitotane administration might represent a promising pharmacological strategy to treat ACC patients, enabling to potentiate mitotane actions while reducing mitotane doses and minimizing potential side effects.

The present study should also facilitate identification of predictive factors of response to mitotane. As previously published, ACAT1 expression, as a protein component of MAM, seems to be a promising predictive factor of response, even though these preliminary observations require further confirmation [5]. FATE1, a cancer-testis antigen expressed in ACC, antagonizes drug-induced apoptosis by uncoupling MAM as recently demonstrated by Doghman-Bougherra *et al* and could also represent a promising factor of resistance to mitotane [29]. Finally, metabolomic studies demonstrate that Aspartate/Glutamate ratio constitutes a specific hallmark of mitotane efficiency and could therefore represent an early marker of response to mitotane, although its potential role remains to be further explored.

In conclusion, our results identify MAM as an important intracellular target of mitotane enabling us to select proteins that could represent predictive factors of response or resistance. Targeting MAM components such as ACAT1 or TSPO associated or not with mitotane could also constitute a novel approach to treat ACC patients.

## MATERIALS AND METHODS

### Human adrenocortical cells and mitochondria isolation

For these *in vitro* studies, H295R cells (from passage 2 to 15) were cultured as previously described [3]. *O,p*’-DDD or mitotane (HRA Pharma) and PK 11195 (Sigma-Aldrich, St. Louis, MO) were solubilized in dimethyl sulfoxide (DMSO, Sigma-Aldrich) and used at the indicated concentrations ranging from 0 to 100 mM. In all experiments, the percentage of DMSO in culture medium never exceeded 0.1% v/v.

Mitochondrial fractions were purified and prepared from permeabilized H295R cells using digitonin and Percoll as previously described [30].

### Metabolomic analysis

Metabolomic investigations were performed by ^1^H high-resolution magic angle spinning (HRMAS) nuclear magnetic resonance (NMR) spectroscopy at Strasbourg University Hospital. Frozen pellets of respectively intact untreated and mitotane-treated H295R cells, were used for NMR-based metabolomic analysis. Each sample (approximately 15 mg/analysis corresponding to about 10^7^ cells) was introduced into a 30 μL disposable insert. 10 μL of D_2_O were added to the rotor to provide a lock frequency for the NMR spectrometer. The exact weight of the sample used was determined by weighting the empty insert and the insert filled by the cellular sample. Inserts containing cells were stored at −80°C and placed in a 4-mm ZrO_2_ rotor just before the HRMAS analysis. HRMAS NMR spectra were recorded on a Bruker Avance III 500 spectrometer operating at a proton frequency of 500.13 MHz and equipped with a 4-mm double resonance (^1^H, ^13^C) gradient HRMAS probe. A Bruker Cooling Unit was used to regulate the temperature by cooling down the bearing air flowing into the probe. To minimize the effects of tissue degradation, all spectra were acquired at a temperature of 4°C. This value was calibrated exactly using a 100% methanol sample. To keep the rotation sidebands out of the spectral region of interest and to minimize sample degradation, all NMR experiments were conducted on samples spinning at 3502 Hz. For each sample, a one-dimensional proton spectrum using a Carr-Purcell-Meiboom-Gill (CPMG) pulse sequence with presaturation of the water signal was acquired. To eliminate signal losses due to B1 inhomogeneity, the inter-pulse delay between the 180° pulses of the CPMG pulse train was synchronized with the sample and set to 285 μs. The number of loops was set to 328, thus giving the CPMG pulse train a total length of 93 ms. The parameters for the CPMG experiment were set as follows: sweep width, 14.2 ppm; number of points, 32k; relaxation delay, 2 s; and acquisition time, 2.3 s. A total of 1024 FIDs (free induction decay) were acquired resulting in an acquisition time of 75 min. Data were zero-filled to a 2k*1k matrix and weighted with a shifted square sine-bell function before Fourier transformation. HRMAS NMR spectra were bucketed into integral regions 0.01 ppm wide (ppm range, 4.8–1) using AMIX 3.8 software (Bruker GmbH, Germany) and exported into SIMCA P (version 11.0, Umetrics AB, Umeå, Sweden). Spectra were referenced by setting the lactate doublet chemical shift to 1.33 ppm. To accommodate the influence of metabolites present at both high and low concentrations, without emphasizing spectral noise, unit variance scaling was employed for all analyses.

Metabolites were assigned using standard metabolite chemical shift tables available in the literature [31]. To confirm resonance assignments, two-dimensional (2D) homonuclear and heteronuclear (^1^H-^13^C) experiments were also recorded immediately after the end of 1D spectra acquisition. Because the long duration of these experiments is potentially responsible of cellular degradation during NMR acquisition, only a few representative samples of both groups (e.g.: treated and untreated cells) were analyzed by 2D experiments. A combination of principal component analysis (PCA) and partial least square discriminant analysis (PLS-DA) was herein adopted [32]. The PCA was performed to evaluate the quality of the data and to identify possible outliers. Then, the PLS-DA was employed to optimize the separation between cells subgroups. Cross-validation was used to determine the number of components and to avoid data overfitting. Fourteen cellular samples (*n* = 7 untreated, *n* = 7 treated by 50 μM of mitotane during 48 h) were analyzed in an exploratory manner. Two measurements of model quality were reported for PLS-DA: R2Y and Q2 representing respectively, the goodness of fit (i.e. data variation) and the goodness of prediction, as estimated by cross-validation. Accordingly, the PLS-DA was performed on the whole set of variables to identify (and afterwards quantify) those metabolites with highest discriminating power. The procedure for metabolite quantification has been previously described [32, 33]. Briefly, quantification was performed using an external reference standard of lactate, scanned under the same analytical conditions. Spectra within the range of 8.65–1 ppm were normalized according to sample weight and peaks of interest were automatically defined. Quantification results were expressed as nmol/mg of cell pellet.

### Lipidomic analysis

The lipidomic analysis was essentially performed as described earlier [34].

Materials: 1,2-dipalmitoyl-sn-glycero-3-phosphocholine-N,N,N-trimethyl-d9 PC 16:0/16:0 d9 (#AVA-860352C), PE 14:0/17:1 (#AVA-LM-1104), PA 14:0/17:1 (#AVA-LM-1404) and PS 14:0/17:1 (#AVA-LM-1304) were used as internal standards. PC 14:0/16:0 (#AVA-850445C), PC 16:0/16:0 (#AVA-850355C), PC 16:0/18:1 (#AVA-850457C), PC 18:0/18:1 (#AVA-850467C), PC 18:0/18:2 (#AVA-850468C), PC 18:0/20:4 (#AVA-850469C), PC 18:0/22:6 (#AVA-850472C), PE 18:1/18:1 (#AVA-850725C), PE 16 :0/18 :1 (#AVA-850757C), PA 16:0/18:1 (#AVA-840857), PA 16 :0/20 :4 (#AVA-840859), PA 16 :0/22 :6 (#AVA-840860), PS 16:0/20:4 (#AVA-840061) and PS 16:0/22:6 (#AVA-840062) were used as calibration standards. All standards were purchased from Coger SAS (Paris, France) distributor of Avanti Polar Lipids (Alabaster, AL, USA). LC/MS grade solvents were used without further purification and obtained from Sigma-Aldrich (St Louis, MO, USA) or VWR (West Chester, PA, USA).

Extraction: Sample was extracted with 0.8 mL acidified methanol (0.1N HCl) containing a mixture of internal standard (358 pmol PC d9, 147 pmol PS 14:0/17:1, 63 pmol PE 14:0/17:1 and 16.3 pmol PA 14:0/17:1) and 0.8 mL chloroform. Phase separation was obtained following addition of a total volume of 0.8 mL aqueous solution. The suspension was vortexed for 1 min and centrifuged at 3600 × *g* for 10 min at 4°C. The lower organic phase was transferred into an amber glass tapered-base vial (#C4000-V2, Thermo Fisher Scientific, MA, USA) and dried in a speed vacuum for 45 min. Phospholipids were reconstituted into 40 μL of LC/MS/MS solvent.

LC/MS/MS analysis: Lipids were quantified by LC-ESI/MS/MS using a QTrap 4000 mass spectrometer (AB Sciex, Framingham, MA, USA) equipped with a turbo spray ion source (300°C) combined with an LC20AD HPLC system, a SIL-20AC autosampler (Shimadzu, Kyoto, Japan) and the Analyst 1.5 data acquisition system (AB Sciex, Framingham, MA, USA).

Quantification of phospholipids and sphingolipids was performed in positive-ion mode (Supplementary Tables 1 and 2). Sample (4 μL) was injected to a Symmetry Shield RP8 3.5 μm 2.1 × 50 mm reverse phase column (Waters Corporation, Milford, MA, USA) using a gradient from 85:15 to 91:9 (v/v) methanol/water containing 5 mM ammonium formate and 0.1% formic acid over 20 min at a flow rate of 0.1 mL/min. Lipid species were detected using multiple reaction monitoring (MRM) reflecting the headgroup fragmentation of each lipid class. PC species were detected as product ions of m/z 184, PE, PS and PA as neutral losses of respectively m/z 141, 185 and 115. N2 was used as a collision gas. Blank and control samples were extracted in parallel with each batch to ensure quality control as described elsewhere [35].

### Western blot analysis

Total protein extracts were prepared and Western blot analyses were performed as previously described [36]. Antibodies used were a rabbit polyclonal anti-VDAC antibody (1:500 dilution, Abcam #15895), a rabbit polyclonal anti-TSPO antibody (1:500 dilution, Abcam #109497) and a rabbit polyclonal anti-PISD antibody (1:500 dilution, Abcam #119960) with a mouse monoclonal anti-α-Tubulin antibody (1:10,000 dilution, Sigma-Aldrich) or a mouse monoclonal anti-DRP1 antibody (1:1,000 dilution, Abcam #56788), a mouse monoclonal anti-CYP11A1 antibody (1:500 dilution, Sigma-Aldrich #09166) and a mouse monoclonal anti-ATAD3A antibody (1:1,000 dilution, Abcam #67992) with a rabbit polyclonal anti-GAPDH antibody (1:10,000 dilution, Sigma-Aldrich). Primary incubation was followed by 1 h incubation at room temperature with secondary antibodies coupled to a fluorochrome, Dylight anti-Rabbit 800 at a dilution of 1:10,000 or Dylight anti-Mouse 680 at a dilution of 1:15,000 (Fischer Scientific). Signal fluorescence intensity was detected and measured by Odyssey Fc (Li-Cor Biotechnologies).

### Immunocytochemistry

H295R cells were seeded on 4-well Tissue Culture Chambers (Sarstedt SARL, Marnay, France). After drug treatment, cells were fixed during 30 min with 4 % paraformaldehyde (PFA) and treated for immunochemistry as previously described [37]. Primary mouse anti-Calnexin antibody (AF18) (Novus Biologicals LLC, Littleton CO, USA) along with the secondary anti-mouse antibody Alexa 555 (Life Technologies, Saint Aubin, France) was used to detect the ER. The anti-COX2 antibody [38] was used with the secondary anti-rabbit antibody Alexa 488 (Life Technologies, Saint Aubin, France) to stain the mitochondria. After primary and secondary antibody labeling, cells were post-fixed during 10 min with 4% PFA. Counter staining of nuclei was performed with 0.5 μg/mL DAPI (4′,6′-diamidino-2-phenylindole). Chambers were removed out and a coverslip were mounted on each slide with ProLong Gold mounting medium (Life Technologie SAS, Saint-Aubin, France). The absence of bleed-through fluorescence in both Green an Red channels was confirmed. (Supplementary Figure 1).

### Deconvolution microscopy and colocalization quantification

Fluorescence deconvolution microscopy was performed with Image Pro Plus AMS (Media Cybernetics Inc, Marlow, UK) using a Mono Q Imaging Retiga 2000R Fast 1394 camera (Q Imaging Inc., Surrey, British Columbia, Canada). Cells were observed and acquired with an automated upright BX61 microscope (Olympus, Rungis, France) at 100x magnification (1.4 NA). A z-series of focal planes was digitally imaged and deconvolved with the 3D blind iterative algorithm (Image Pro Plus AMS) to generate high-resolution images. The image processing package Fiji [39] was used to quantify the colocalization of COX2 and Calnexin. Calculation of Manders' Colocalization Coefficients (MCC) and Pearson's Correlation Coefficient (PCC) [19] were performed using Coloc 2 a Fiji's plugin for standardized colocalization (http://imagej.net/Coloc_2). Briefly, 3D image stacks were converted in 8-bits images and a background subtraction of 50 was applied for each picture (rolling ball radius). Both pictures were then processed by the coloc2 plugin and PCC/MMC values were exported to Excel.

### Apoptosis analysis

Apoptosis tests were performed by using the Caspase-Glo 3/7 assay (Promega, Madison, WI) according to the manufacturer's recommendations. Cells were cultured in 96-well plates and treated with 0 to 100 μM o,p’-DDD for 24 h ± 50 μM PK11195. Luminescence was measured 1 h after addition of Caspase-Glo 3/7 solution (equal volume) by luminometry (Viktor, Perkin Elmer).

### Cortisol secretion

H295R cells seeded 6-well plates, were incubated for 48 h with vehicle (DMSO, control group); 50 μM PK 11195; 10 μM mitotane (o,p’DDD) or a combination of both PK 11195 (50 μM) + o,p’DDD (10 μM). After 48 h incubation, 500 μL cell culture supernatants were centrifuged at 1,500 rpm for 3 min to remove cell debris, and 250 μL of the corresponding supernatants were immediately used for steroid measurements. An LC-MS/MS method recently established [40] allows us to precisely quantify cortisol levels (with a limit of quantification at 1 ng/mL or 2.7 nmol/L).

### Statistical analysis

Results are expressed as means ± SEM of n independent replicates performed in the same experiment or from separate experiments (n). Non-parametric Mann Whitney *U*-tests were used when appropriate and differences between groups were analyzed using non-parametric Kruskall-Wallis multiple comparison tests followed by a post-test of Dunn's (Prism software, GraphPad, CA). A *P* value of 0.05 was considered as statistically significant (*P < 0.05; **P < 0.01; ***P < 0.001).

## SUPPLEMENTARY MATERIALS FIGURES AND TABLES


