# Supplementary material for: Identifying mitotane-induced mitochondria-associated membranes dysfunctions: metabolomic and lipidomic approaches
Source: Oncotarget. 2017 Jul 4;8(66):109924–40. doi: 10.18632/oncotarget.18968 (PMC5746354; doi:10.18632/oncotarget.18968)
Supplement: Supplementary file 1 [file oncotarget-08-109924-s001.pdf]

Identifying mitotane-induced mitochondria-associated membranes dysfunctions: metabolomic and lipidomic approaches

SUPPLEMENTARY MATERIALS

Supplementary Table 1: MS ionization conditions

Lipid Class	Parent Ion	MS Experiment	DP	EP	CE	CXP
Phosphatidylcholine (PC)	$[M+H]^+$	PIS 184m/z	116	10	45	12
Phosphatidylethanolamine (PE)	$[M+H]^+$	NL 141m/z	114	10	44	15
Phosphatidylserine (PS)	$[M+H]^+$	NL 185m/z	111	10	29	20
Phosphatidic acid (PA)	$[M+NH4]^+$	NL 115m/z	96	10	21	22

PIS: product ion scan, NL: neutral loss, DP: declustering potential, EP: entrance potential, CE: collision energy, CXP: cell exit potential.

Supplementary Table 2: List of analyzed phospholipids

PA(32:0)	PC(34:3)	PE(34:3)	PS(34:1)
PA(32:1)	PC(36:1)	PE(36:0)	PS(34:2)
PA(34:1)	PC(36:2)	PE(36:1)	PS(34:3)
PA(34:2)	PC(36:3)	PE(36:2)	PS(36:0)
PA(36:1)	PC(36:4)	PE(36:3)	PS(36:1)
PA(36:2)	PC(36:5)	PE(36:4)	PS(36:2)
PA(36:3)	PC(38:3)	PE(36:5)	PS(36:3)
PA(36:4)	PC(38:4)	PE(36:6)	PS(36:4)
PA(38:3)	PC(38:5)	PE(38:0)	PS(38:0)
PA(38:4)	PC(38:6)	PE(38:1)	PS(38:1)
PA(38:5)	PC(40:2)	PE(38:2)	PS(38:2)
PA(38:6)	PC(40:3)	PE(38:3)	PS(38:3)
	PC(40:4)	PE(38:4)	PS(38:4)
PC(28:0)	PC(40:5)	PE(38:5)	PS(38:5)
PC(30:0)	PC(40:6)	PE(38:6)	PS(38:6)
PC(30:1)	PC(40:7)	PE(40:4)	PS(40:3)
PC(32:0)	PC(40:8)	PE(40:5)	PS(40:4)
PC(32:1)		PE(40:6)	PS(40:5)
PC(32:2)	PE(32:1)	PE(40:7)	PS(40:6)
PC(34:0)	PE(34:0)	PE(42:5)	PS(40:7)
PC(34:1)	PE(34:1)	PE(42:6)	
PC(34:2)	PE(34:2)	PE(42:7)	

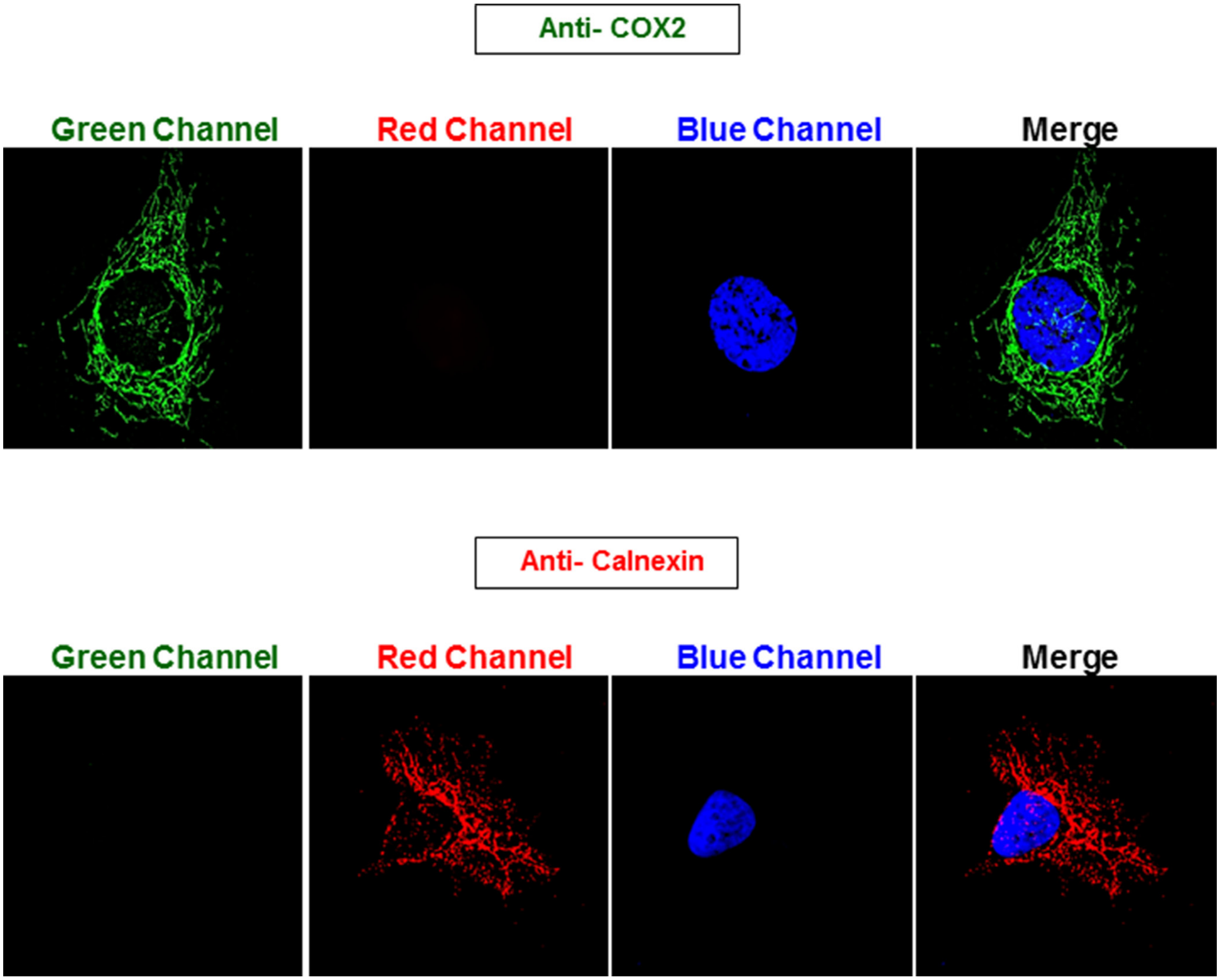

Supplementary Figure 1: Control bleed-through of fluorescence emission into channels. H295R cells were fixed and analyzed by immunocytochemistry as described in Materials and Methods section. Either the anti-COX2 antibodies (Green staining) or the anti-Calnexin antibody (Red staining) were used separately to detect the mitochondria or the endogenous endoplasmic reticulum, respectively (see Material and method section). First antibody was followed by a 45 min incubation with a mix of the two corresponding secondary antibodies, anti-rabbit Alexa 488 (green) and anti-mouse Alexa 555 (red). DAPI staining delineates the nuclei. A z-series of focal planes was digitally imaged and deconvolved with a 3D blind iterative algorithm to generate high-resolution images. Note the lack of bleed-through fluorescence in both channels.

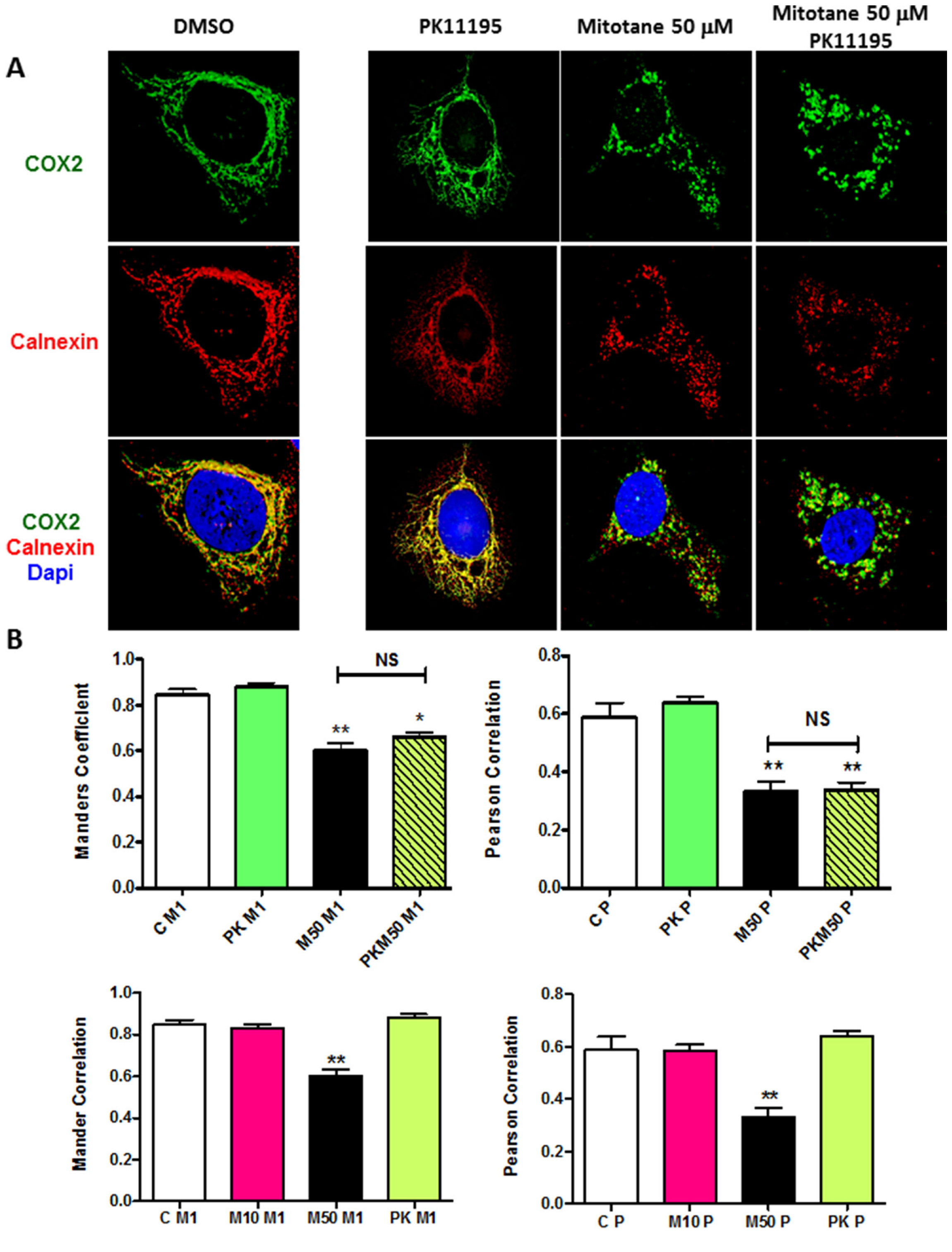

Supplementary Figure 2: PK11195 potentiates mitotane-induced MAM disruption. (**A**) H295R cells were incubated for 48 h with DMSO (0.1% v/v) or 50 µM mitotane in the absence or presence of PK11195 (50 µM). After drug treatment, cells were fixed and analyzed by immunocytochemistry as described in legend of Figure 6. The anti-COX2 (green) and anti-calnexin antibodies (red) were used to detect the mitochondria and ER, respectively. DAPI staining delineates the nuclei. A z-series of focal planes was digitally imaged and deconvolved with a 3D blind iterative algorithm to generate high-resolution images. DMSO panel used in Figure 7 was used as the control condition. In the presence of 50 µM mitotane the mitochondrial interconnected tubular network is readily disorganized and the mitochondrial filamentous aspect dramatically disappears to be replaced by a punctiform network. No clear-cut visual differences are observed when mitotane is used in combination with PK11195. (**B**) Manders' Colocalization Coefficients (MCC) and Pearson Correlation Coefficient (PCC) quantification, are shown to quantify colocalization of the two proteins for each condition, DMSO (**C**) mitotane (M), PK11195 (P), respectively. ($n > 5$). $^{*}P < 0.05$ and $^{**}P < 0.01$, Mann-Whitney U-tests.
